# Formation polarity dependent improved resistive switching memory characteristics using nanoscale (1.3 nm) core-shell IrO_x _nano-dots

**DOI:** 10.1186/1556-276X-7-194

**Published:** 2012-03-22

**Authors:** Writam Banerjee, Siddheswar Maikap, Chao-Sung Lai, Yi-Yan Chen, Ta-Chang Tien, Heng-Yuan Lee, Wei-Su Chen, Frederick T Chen, Ming-Jer Kao, Ming-Jinn Tsai, Jer-Ren Yang

**Affiliations:** 1 Department of Electronic Engineering, Chang Gung University, 259 Wen-Hwa 1st Rd., Kwei-Shan, Tao-Yuan 333, Taiwan; 2 Department of Materials Science and Engineering, National Taiwan University, Taipei 106, Taiwan; 3 Material and Chemical Research Laboratories, Industrial Technology Research Institute, Hsinchu, Taiwan 310, Taiwan; 4 Electronic and Opto-Electronic Research Laboratories, Industrial Technology Research Institute, Hsinchu 310, Taiwan

**Keywords:** Resistive switching, Nanoscale, Memory, IrO_x _nano-dot, Formation polarity

## Abstract

Improved resistive switching memory characteristics by controlling the formation polarity in an IrO_x_/Al_2_O_3_/IrO_x_-ND/Al_2_O_3_/WO_x_/W structure have been investigated. High density of 1 × 10^13^/cm^2 ^and small size of 1.3 nm in diameter of the IrO_x _nano-dots (NDs) have been observed by high-resolution transmission electron microscopy. The IrO_x_-NDs, Al_2_O_3_, and WO_x _layers are confirmed by X-ray photo-electron spectroscopy. Capacitance-voltage hysteresis characteristics show higher charge-trapping density in the IrO_x_-ND memory as compared to the pure Al_2_O_3 _devices. This suggests that the IrO_x_-ND device has more defect sites than that of the pure Al_2_O_3 _devices. Stable resistive switching characteristics under positive formation polarity on the IrO_x _electrode are observed, and the conducting filament is controlled by oxygen ion migration toward the Al_2_O_3_/IrO_x _top electrode interface. The switching mechanism is explained schematically based on our resistive switching parameters. The resistive switching random access memory (ReRAM) devices under positive formation polarity have an applicable resistance ratio of > 10 after extrapolation of 10 years data retention at 85°C and a long read endurance of 10^5 ^cycles. A large memory size of > 60 Tbit/sq in. can be realized in future for ReRAM device application. This study is not only important for improving the resistive switching memory performance but also help design other nanoscale high-density nonvolatile memory in future.

## Introduction

Many oxide materials such as NiO_x _[1-4], HfO_x _[5,6], Cu_2_O [7], Gd_2_O_3 _[8], TaO_x _[9,10], TiO_2 _[11], ZrO_2 _[12,13], AlO_x _[14-16], Na_0.5_Bi_0.5_TiO_3 _[17], SrTiO_3 _[18], and so on have been reported for nanoscale nonvolatile resistive switching random access memory (ReRAM) applications. Basically, a single layer of resistive switching material has been investigated by many groups. Due to stochastic nature of the conducting filament in a single binary-oxide resistive switching material [19], it results poor switching cycles. To improve the ReRAM device performance, many nano-dots (NDs) such as ruthenium (Ru) [19], gold (Au) [20], copper (Cu) [21], nickel [22], and so on have been reported. External electric field will control conducting filament formation/rupture through the NDs in the same pathway [22], which can improve the switching parameters. However, it is still an issue to improve the resistive switching parameter using NDs in the insulating materials. Even though many types of NDs have been reported to improve the resistive switching memory characteristics, the core-shell iridium-oxide (IrO_x_) NDs embedded in high-κ Al_2_O_3 _film for IrO_x_/Al_2_O_3_/IrO_x_-NDs/Al_2_O_3_/WO_x_/W ReRAM device have not been reported yet. The electrically conducting iridium (Ir) or iridum-oxide (IrO_x_) metal is an attractive one due to its high work function (> 5 eV) and noble metal. Furthermore, formation polarity is also one of the important keys to improve the resistive switching performance because it can also control the conducting pathways through the NDs. It is known that the Gibbs free energies of the Al_2_O_3_, IrO_2_, WO_3_, and WO_2 _films are -1,582.3 [23], -183.75, -506.63, and -526.0 kJ/mole, respectively, at 300 K [24]. It is suggested that the high-κ Al_2_O_3 _film will be easily oxidized than those of the WO_3_, WO_2_, and IrO_2 _films. Under external bias, the oxygen ions (O^2-^) will be generated from the Al_2_O_3 _layer, while oxygen vacancies (V_o_) will be supplied from the WO_x _layer. It is known that the Al_2_O_3 _film has negative-type defects. The defects density in the Al_2_O_3 _film will be increased by including core-shell IrO_x_-NDs. It is expected that the core-shell IrO_x_-NDs embedded in the Al_2_O_3 _films will not only increase the defect density but also will guide the conducting filament through a single nano-dot, which can be controlled also by external formation polarity. Due to noble metal of Ir, it will not be oxidized under external bias; however, the surface of the IrO_x_-ND will control the oxygen-vacancy filament. Even the IrO_x _metal, it will also not be oxidized under external bias. Due to IrAlO mixture on the IrO_x_-ND surface, this will control the filament formation/rupture. In order to understand the defective IrO_x_-NDs embedded in the Al_2_O_3 _films, the memory capacitors in an IrO_x_/Al_2_O_3_/IrO_x_-ND/Al_2_O_3_/SiO_2_/n-Si structure have also been reported. Improved resistive switching parameters such as *set*/*reset *voltages, low resistance state (LRS)/high resistance state (HRS), switching cycles, read endurance of > 10^5 ^times, and extrapolated 10 years data retention at 85°C of the IrO_x_-ND ReRAM device under positive formation polarity (PF) have been reported. Formation polarity dependent resistive switching phenomena have been also explained by oxygen ions migration under external bias. The ReRAM device with layer-by-layer has been observed by high-resolution transmission electron microscopy (HRTEM), energy dispersive X-ray spectroscopy (EDX), and X-ray photoelectron spectroscopy (XPS) analyses.

## Experimental

**Figure 1:**
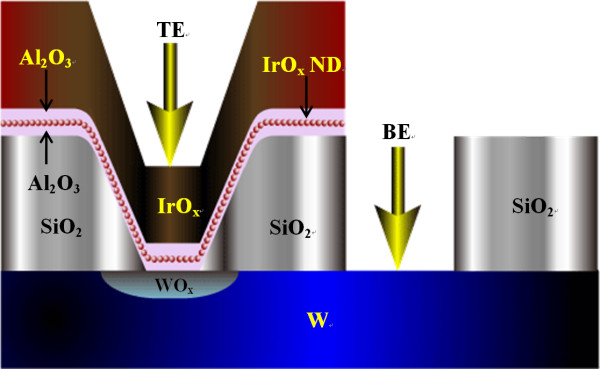
**The schematic view of the fabricated IrO_x_-ND based ReRAM devices**.

Resistive switching memory characteristics using IrO_x_-NDs in an IrO_x_/Al_2_O_3_/IrO_x_-NDs/Al_2_O_3_/WO_x_/W metal-insulator-metal structure were investigated. First, tungsten (W) bottom electrode (BE) with a thickness of approximately 100 nm was deposited by sputtering on SiO_2 _(200 nm)/Si substrate. To design the ReRAM device, the SiO_2 _layer with a thickness of approximately 150 nm was deposited. Different via sizes from 0.6 × 0.6-8 × 8 μm^2 ^were patterned using standard lithography. Then, photoresist was coated and opened the active and top electrode (TE) regions. Then, high-κ Al_2_O_3 _film with a thickness of approximately 3 nm was deposited by radio frequency (rf) sputtering system using Al_2_O_3 _target. Then, the IrO_x _nano-layer with a nominal thickness of approximately 1.5 nm was also deposited by rf sputtering system. The Ir target was used during deposition of the IrO_x _layer. The ratio of argon (Ar) to oxygen (O_2_) gasses was 1:1 (i.e., 25 sccm:25 sccm) during deposition. Then, high-κ Al_2_O_3 _film with a thickness of approximately 3 nm was deposited using the same conditions above. Finally, the IrO_x _metal as a TE with a thickness of approximately 350 nm was deposited. The resistivity of the IrO_x _metal layer was approximately 600 μΩ cm, which is similar to reported result (approximately 500 μΩ cm) of IrO_2 _[25]. This suggests that the IrO_x _is a metallic film. During deposition of the Al_2_O_3 _and IrO_x _nanolayers, the bottom W electrode was partially oxidized, which results in a WO_x _film on the W surface. To fabricate the ReRAM device, a lift-off process was used. Schematic view of our ReRAM device is shown in Figure 1. Microstructure of all layers in the ReRAM device was investigated by HRTEM. Material compositions were studied by XPS with Al K_α _monochrom X-ray and energy of 1,486.6 eV. X-ray with a small-diameter of 500 μm was focused on IrO_x_/Al_2_O_3_/WO_x_/W structure. The surface spectra were taken by etching the layers one-by-one. All spectra were calibrated by C1s spectrum at an energy of 284.6 eV. Resistive switching memory characteristics were measured by HP 4156C semiconductor parameter analyzer {Agilent Technologies, 5301 Stevens Creek Blvd, Santa Clara, CA, 95051, USA} for a device size of 0.6 × 0.6 μm^2^.

Furthermore, charge-trapping behaviors of the IrO_x_-NDs in an IrO_x_/Al_2_O_3_/IrO_x_-NDs/Al_2_O_3_/SiO_2_/n-Si metal-insulator-semiconductor (MIS) structure were also investigated. After cleaning the n-type Si (100) wafers, a SiO_2 _tunneling oxide with a thickness of approximately 3.6 nm was grown. Then, high-κ Al_2_O_3 _tunneling oxide with a thickness of approximately 1.2 nm was deposited. So the thickness of stack tunneling oxide (SiO_2 _+ Al_2_O_3_) was approximately 4.8 nm. The IrO_x _metal gate electrode with a gate area (Φ) of 3.14 × 10^-4 ^cm^2 ^was deposited by a shadow mask. Capacitance-voltage (C-V) hysteresis characteristics of the MIS structure were measured by HP 4284A LCR meter {Agilent Technologies, 5301 Stevens Creek Blvd, Santa Clara, CA, 95051, USA}.

## Results and discussion

**Figure 2:**
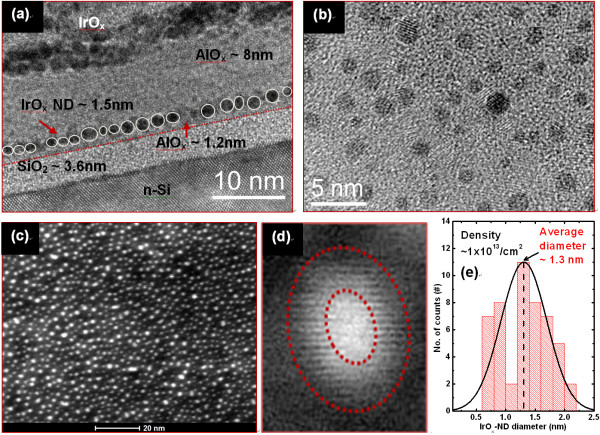
**Different images of IrO_x_-NDs in an IrO_x_/Al_2_O_3_/IrO_x_-NDs/Al_2_O_3_/SiO_2_/n-Si MIS structure**. (**a**) Cross-sectional HRTEM image on IrO_x_-NDs in an IrO_x_/Al_2_O_3_/IrO_x_-NDs/Al_2_O_3_/SiO_2_/n-Si MIS structure. (**b**) Plane view TEM image. (**c**) Scanning tunneling electron microscope image and (**d**) a single core-shell IrO_x_-ND. (**e**) Histogram of the IrO_x_-NDs with an average size of 1.3 nm with a high density of IrO_x_-NDs of 1 × 10^13^/cm^2^.

**Figure 3:**
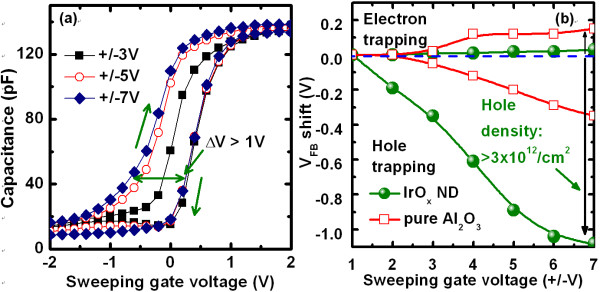
**Typical hysteresis characteristics of the IrO_x_-ND MIS capacitors and V_FB _shift with sweeping gate voltages**. (**a**) Typical C-V hysteresis characteristics of the IrO_x_-ND MIS capacitors and (**b**) V_FB _shift with sweeping gate voltages for the IrO_x_-ND and pure Al_2_O_3 _charge-trapping devices.

Figure 2a shows the size and density of the IrO_x_-NDs in MIS structure. The thickness of Al_2_O_3 _blocking oxide is 8 nm. As-deposited IrO_x _nanolayer shows self-assembly NDs because as-deposited layer is very thin (1.5 nm). Isolated IrO_x_-NDs are observed clearly (Figure 2b). The IrO_x_-NDs have high density of approximately 1 × 10^13^/cm^2 ^(Figure 2c), which results a large memory size of > 60 Tbit/sq in. The nano-dots are core-shell structure (Figure 2d). Core region of the NDs is Ir-rich, and shell region is oxygen-rich or IrAlO mixture. It is indicated that V_o _can be observed in the core region, and oxygen accumulations (O^2-^) can be observed in the shell region. The IrO_x_-NDs have a nanoscale average diameter of approximately 1.3 nm (Figure 2e). The hole or electron trapping can be measured from the C-V hysteresis characteristics of the MIS capacitors. Figure 3a shows high-frequency (1 MHz) clockwise C-V hysteresis characteristics of the MIS capacitors. A small equivalent oxide thickness of approximately 8 nm is obtained. A large memory window of approximately 1.2 V is observed under a sweeping gate voltage of ± 7 V for the IrO_x_-ND device, while a small memory of 0.3 V is observed for the pure Al_2_O_3 _chrage-trapping layers (Figure 3b). The neutral flat-band voltage (V_FB_) is found to be +0.15 V for the IrO_x_-ND devices (not shown here). The hole and electron trapping densities can be calculated using equation in reference [26]. High hole-trapping densities for the pure Al_2_O_3 _and IrO_x_-NDs capacitors are found to be approximately 5.6 × 10^11^/cm^2 ^and approximately 3.1 × 10^12^/cm^2^, respectively (Figure 3b). Almost no electrons are trapped in the core-region IrO_x_-ND under positive voltage, but holes are trapped on the shell region under negative bias on the IrO_x _electrode. This suggests that the holes can be trapped on the nano-dot surface easily because the surface has negative-type defects. These trapping holes as well as oxygen vacancy on the surface of the NDs will lead the conducting filament as well as resistive switching memory parameters under formation polarity, which have been explained below.

**Figure 4:**
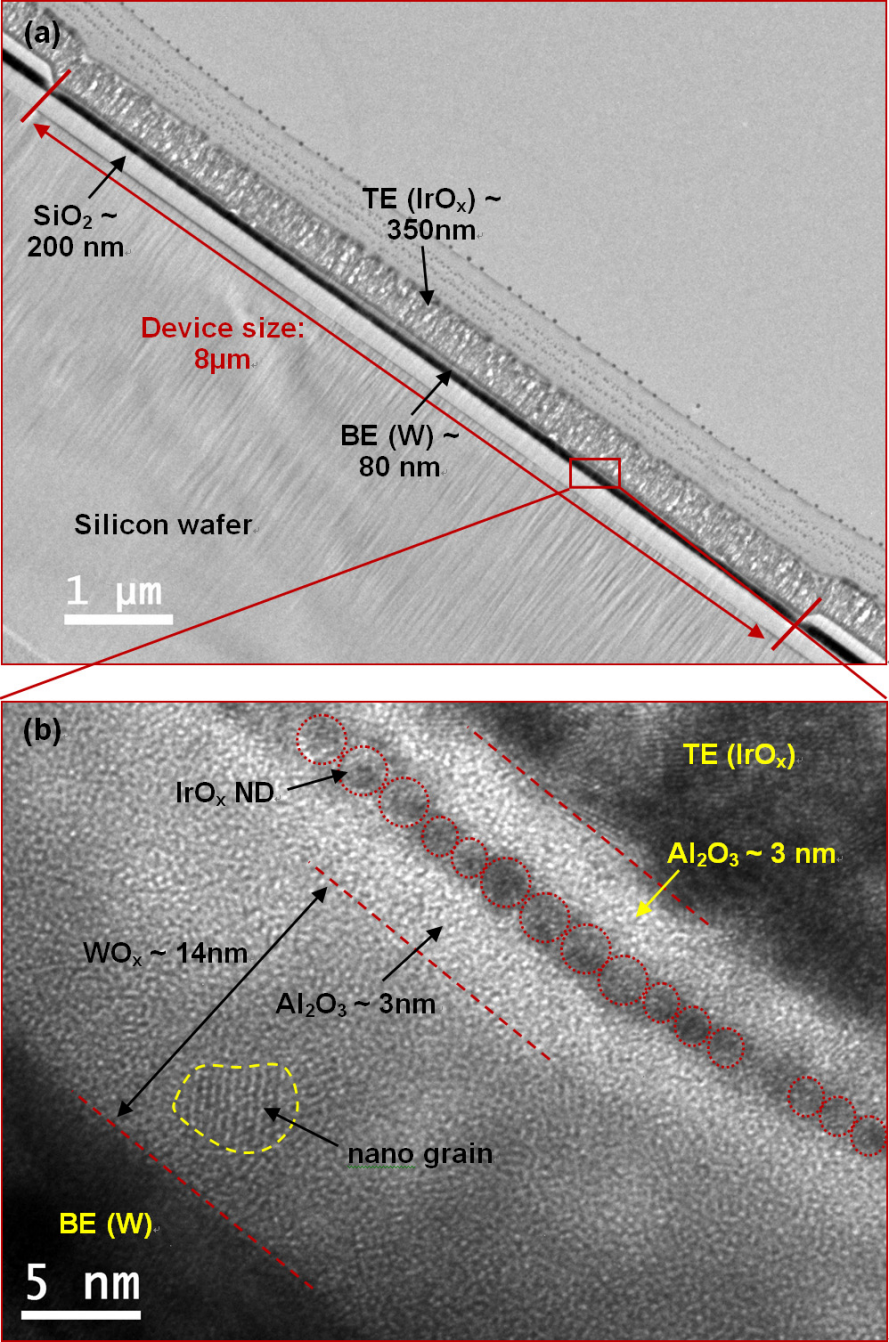
**The cross-sectional TEM and HRTEM images**. (**a) **Cross-sectional TEM image of an IrO_x_/Al_2_O_3 _/IrO_x_-NDs/Al_2_O_3_/WO_x_/W ReRAM device with a device size of 8 × 8 μm^2^. (**b) **HRTEM image of the stack layers.

Figure 4a shows typical HRTEM image of a ReRAM device with a size of 8 × 8 μm^2^. The stack switching layers with self-assembly IrO_x_-NDs are observed clearly in Figure 4b. The IrO_x_-NDs with a thickness of approximately 1.5 nm are sandwiched in between high-κ Al_2_O_3 _layers with a thickness of approximately 3 nm. A small size of approximately 1.3 nm in a diameter is observed. The IrO_x_-NDs shows crystalline, while the high-κ Al_2_O_3 _film shows amorphous. The WO_x _layer with a thickness of approximately 14 nm is also observed, which is expected from our deposition. The WO_x _film shows polycrystalline. All layers are also confirmed by XPS analysis below.

**Figure 5:**
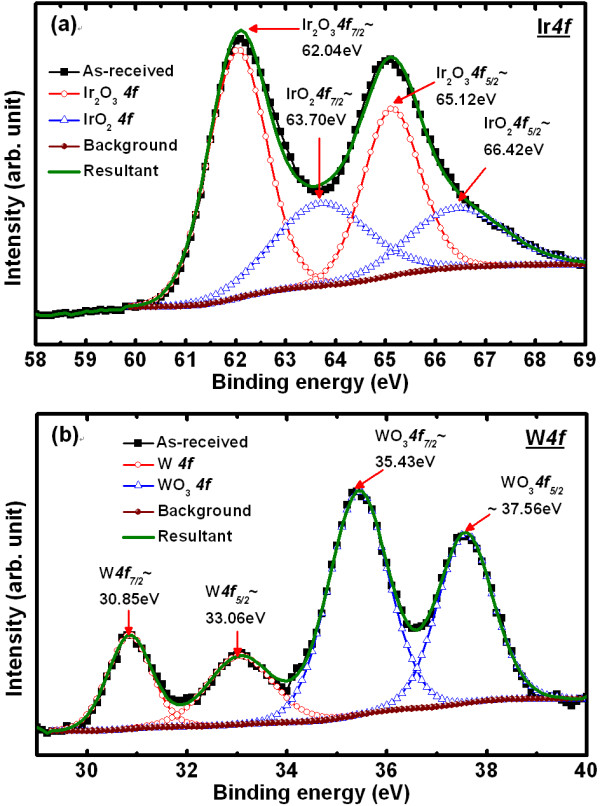
**XPS of Ir*4f *electrons from the IrO_x_-NDs and W*4f *core-level electrons in the WO_x _layer**. (**a**) XPS of the Ir*4f *electrons from the IrO_x_-NDs and (**b**) W*4f *core-level electrons in the WO_x _layer.

Figure 5a shows the XPS spectra of the Ir*4f *core levels with the Ir_2_O_3 _and IrO_2 _compositions. The peak fitting was performed using Shirley background subtraction and Gaussian-Lorentzian functions. Two peaks positions of the X-ray photo-electron spectra are strong indication of the presence of Ir_2_O_3 _*4f*_7/2 _and Ir_2_O_3 _*4f*_5/2 _spin orbital components with peak energies centered at approximately 62.04 and approximately 65.12 eV, respectively. The peak binding energies of the IrO_2 _*4f*_7/2 _and IrO_2 _*4f*_5/2 _doublets are centered at approximately 63.70 and approximately 66.42 eV, respectively. The IrO_2 _peak intensity is smaller than that of the Ir_2_O_3_, which is evidently prove the presence of IrO_x _NDs. This IrO_x_-ND is embedded in the high-κ Al_2_O_3 _films, which is also confirmed by Al*2p *and O*1s *signals (not shown here). Choi et al. [27] reported the peak energy positions for IrO_2_*4f_7/2 _*and IrO_2_*4f_5/2 _*which are located at 61.9 and 64.9 eV, respectively. The presence of metallic W and oxygen-rich WO_3 _can be confirmed by XPS analysis of W*4f *spectra (Figure 5b) in 14 nm-thick WO_x _layer. The peak binding energies of the W*4f*_7/2 _and W*4f*_5/2 _electrons are centered at 30.85 and 33.06 eV, respectively. The energy peak separation is 2.2 eV. The positions of binding energies for WO_3_*4f_7/2 _*and WO_3_*4f_5/2 _*electrons are located at 35.43 and 37.56 eV, respectively, and these values are similar to the reported results at 35.0 and 37.6 eV for the WO_3_*4f*_7/2 _and WO_3_*4f*_5/2 _electrons, respectively [28]. The W sub-oxide (WO_x_) located at 35.0 eV could be formed during the deposition process, and the oxygen-vacancy could be found in the WO_x _films. Due to both the IrO_x_-NDs in the high-κ Al_2_O_3_/WO_x _bilayer structure and high charge-trapping density, improved resistive switching memory performance has been explained below.

**Figure 6:**
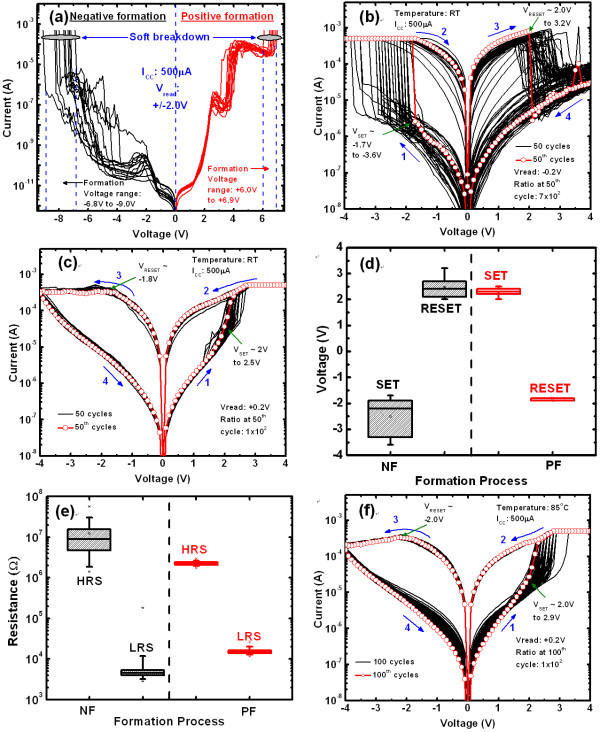
**Formation characteristics of the devices under NF and PF, and I-V characteristics for NF and PF devices**. (**a) **Formation characteristics of the pristine resistive memory devices under NF and PF. (**b**) Typical I-V hysteresis characteristics for the NF devices at RT. (**c**) Typical I-V characteristics with stable switching for the PF devices at RT. Statistical distribution of the NF and PF devices for (**d**) set/reset voltages and (**e) **LRS/HRS. (**f**) Consecutive 100 DC cycles at 85°C for the PF devices.

Figure 6a shows electrochemical formation process of our IrO_x_/Al_2_O_3_/IrO_x_-NDs/Al_2_O_3_/WO_x_/W ReRAM device. The pristine devices show low leakage current. The currents increase with increasing voltage, and it is limited by current compliance (I_CC_) of 500 μA. The average leakage current under negative formation (NF) is 229.7 pA at a read voltage (V_read_) of -2.0 V, while it is 673.3 pA under PF at a V_read _of +2.0 V. Asymmetry values of the leakage currents under NF and PF polarities are quite reasonable due to the different work function (Φ_m_) values of IrO_x _[(Φ_m_)_IrOx _≈ 5.2 eV [14], and of W [(Φ_m_)_W _≈ 4.3-4.91 eV [29]] electrodes. Considering the electron affinities (*χ*) of 3.33 eV [30] for WO_3 _and 1.25 eV for Al_2_O_3 _layers, the values of the effective barrier heights (Φ_b_) are found to be [Φ_b _= (Φ_m_)_W_-(*χ*)_WO3_] 1.3 eV and [(Φ_b_) = (Φ_m_)_IrOx_-(*χ*)_Al2O3_] 3.95 eV, respectively. So the electron injection at the IrO_x_/Al_2_O_3 _interface will be lower than that of the W/WO_x _interface. In consequence, the currents under PF increase rapidly which results a lower and tight distribution of formation voltages as compared to NF (6-7 V vs. -7 to -9 V). This suggests that lower formation voltage can protect device degradation [22]. During the formation process, the high electric field breaks the Al_2_O_3 _film and forms the Al-O bonds with oxygen vacancy as well as oxygen vacancy filament. In consequence, oxygen ions (O^2-^) will be released from the conduction channels. These oxygen ions will move from the Al_2_O_3_/IrO_x_-NDs/Al_2_O_3 _stacks toward WO_x _layer under NF because the TE has negative polarity, and the BE is grounded during measurement. Under PF, the oxygen ions will move toward the TE because the TE has positive polarity. In this case, the oxygen ions will be accumulated at the Al_2_O_3_/TE interface, i.e., oxygen-rich AlO_x _film. So the oxygen vacancy filament can be formed in the Al_2_O_3_/IrO_x_-NDs/Al_2_O_3 _stack. The WO_x _layer will behave as a conductiong layer under PF. After the first reset operation, the ReRAM devices under both NF and PF show bipolar resistive switching characteristics. Figure 6b shows typical I-V characteristics with 50 DC cycles at room temperature (RT), as shown by arrows: 1→4 after NF. The LRS currents are fitted with ohmic, and the HRS currents are fitted with Schottky emission (not shown here). Large variations of the set and reset voltages at a V_read _of -0.2 V are -1.7 to -3.6 V and +2.0 to +3.2 V, respectively (Figure 6d) and large variations in LRS and HRS are also observed (Figure 6e). Typical resistance ratio under NF is approximately 10^3^. Under PF, excellent DC I-V switching cycles are observed (Figure 6c). A tight distribution of the set voltages from +2.0 to +2.9 V as well as reset voltages is observed (Figure 6d). Average HRS and LRS values are approximately 2 MΩ and 20 kΩ, respectively (Figure 6e). So an acceptable resistance ratio of approximately 10^2 ^is obtained under PF. Even at high temperature of 85°C, excellent consecutive 100 DC switching cycles is observed under PF (Figure 6f). To investigate the current conduction mechanism under PF, I-V curves of LRS and HRS are fitted linearly in log-log plot, which are in agreement with the trap-charge controlled space charge limited current model for both LRS and HRS (not shown here).

**Figure 7:**
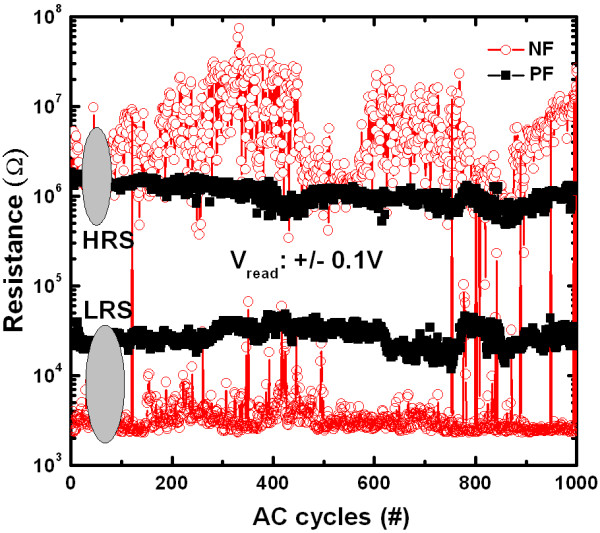
**Improved AC endurance characteristics are observed for the PF devices**.

**Figure 8:**
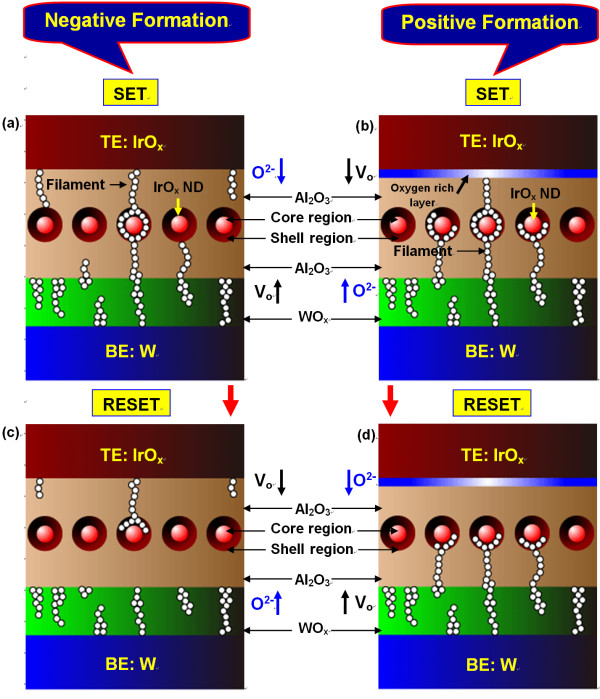
**The schematic illustration of the switching mechanisms of NF and PF devices**. Schematic illustration of the switching mechanisms of (**a**) the NF devices and (**b**) the PF devices under set operation, (**c**) the NF devices and (**d**) the PF devices under reset operation.

Figure 7 shows typical AC endurance characteristics for both NF and PF devices up to 10^3 ^cycles. Both the devices are programmed at a voltage of ± 4 V, a low I_CC _of 500 μA, and a V_read _of ± 0.1 V. Inferior AC cycles are observed for the NF devices, where as the PF devices show excellent switching stability. Improved resistive switching parameters of the IrO_x_/Al_2_O_3_/IrO_x_-NDs/Al_2_O_3_/WO_x_/W structure under PF can be explianed as follows. For the NF devices, the oxygen ions will move from the Al_2_O_3_/IrO_x_-NDs/Al_2_O_3 _stack toward the WO_x _layer under a negative voltage (-V < V_set_) on the TE or the oxygen vacancies will move from the WO_x _layer toward the Al_2_O_3_/IrO_x_-NDs/Al_2_O_3 _stack (Figure 8a). It seems that the oxygen ions will be hidden in to the WO_x _layer. It results the oxygen vacancy filament through the IrO_x_-NDs as well as LRS. Under reset operation (+V > V_reset_), the oxygen ions will move from WO_x _layer toward Al_2_O_3_/IrO_x_-NDs/Al_2_O_3 _stack and oxidize the conducting filament as well as HRS (Figure 8c). Due to the lower barrier height at the W/WO_3 _interface, the injected electrons will be higher. This result indicates a higher O^2-^ions migration and randomized rupture of the conducting filaments. According to our previous report, improved resistive switching characteristics of the IrO_x_-ND ReRAM devices are observed as compared to the pure Al_2_O_3 _ReRAM device under NF due to oxygen-vacancy filament confinement by the IrO_x_-NDs [31]. Even though IrO_x_-NDs are embedded in the Al_2_O_3 _switching material, but higher O^2-^ions migration will reset HRS dispersion resulting a variation of switching cycles. This suggests that the controllable reset operation is also a crucial issue to improve the repeatable switching cycles. So oxygen ions migration leads to the filament formation/rupture in the Al_2_O_3_/IrO_x_-NDs/Al_2_O_3 _stack under NF, which results an inferior switching cycles. In previous literature, similar phenomena using oxygen vacancies generation and recombination to form/rupture a conducting filament to switch the LRS and HRS are also reported in some other ReRAM devices [32,33]. For the PF devices, the oxygen ions will move from the Al_2_O_3_/IrO_x_-NDs/Al_2_O_3 _stack toward the Al_2_O_3_/TE interface under a positive voltage (+V > V_set_) on the TE or the oxygen vacancies will move from the Al_2_O_3_/TE interface (oxygen-rich AlO_x_) toward Al_2_O_3_/IrO_x_-NDs/Al_2_O_3 _stack and sets LRS (Figure 8b). This suggests that an insulating layer as a series resistor with LRS will be created at the Al_2_O_3_/TE interface under set operation, which will limit electron injection at the W/WO_x _interface. This result indicates a thinner and stable conducting filament formation, as shown in Figure 8b. So the LRS of the PF devices is higher than that of the NF devices (Figure 6e) due to series insulator (oxygen-rich AlO_x _layer). Under reset operation (-V < V_reset_), the filament can be ruptured by oxygen ions migration from the Al_2_O_3_/TE (i.e., oxygen-rich AlO_x _layer) and oxidize the partial filament, which results also a stable HRS because the electron injection (O^2- ^ions migration from oxygen-rich AlO_x _layer) from the TE is very low, and this will be limited by the IrO_x_-ND/Al_2_O_3_/TE region, as shown in Figure 8d. The conduction filaments in the WO_x_/Al_2_O_3_/IrO_x_-NDs region will remain after reset operation. Due to this remaining filament, the next set operation will be also easier. Due to this stable reset, repeatable switching cycles are expected for the PF devices. So the HRS of the PF devices is lower as compared to the NF devices (Figure 6e), due to shorter length filament oxidized. Basically, the limitation of electron injections (O^2- ^ions migration) can control the charge trapping (filament oxidation)/de-trapping (filament formation) through the IrO_x_-ND boundary. A similar study has been reported by other group [34]. For the PF devices, the filament can be formed or ruptured repeatedly than that of the NF devices. This reveals that the filament formation/rupture is more controllable (i.e., localized filament) when it is formed at the IrO_x_-NDs/oxygen-rich AlO_x _interface region because it has short length, and an oxygen-rich AlO_x _layer is in series which can protect the higher electron injection. So the improved resistive switching characteristics are expected for the PF devices. It is noted that a small size (1.3 nm) NDs are observed, and the filament diameter can be adjusted through a single ND in future. It indicates that the device size can be down scale to 1.3 nm in future for advanced high-density nonvolatile memory applications.

**Figure 9:**
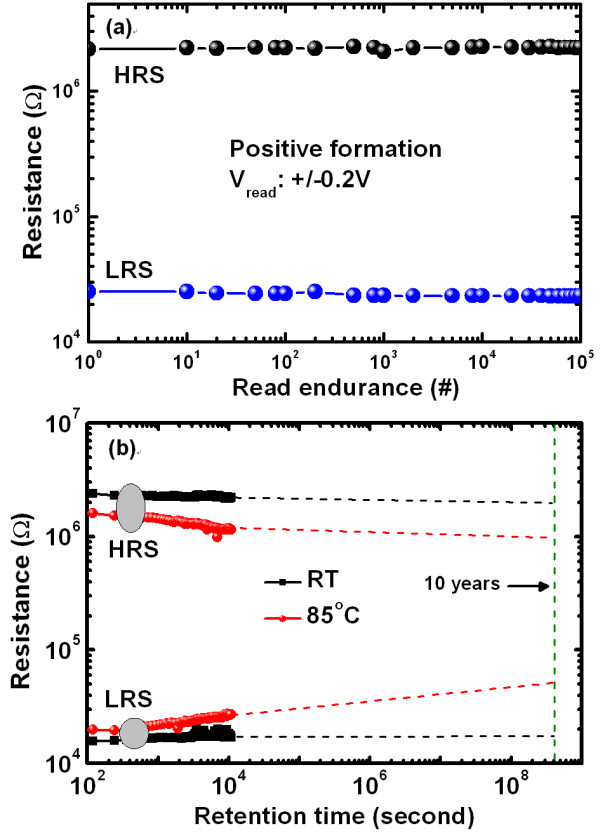
**The non-destructive read endurance of 10^5 ^cycles and retention characteristics of the PF devices**. (**a**) Non-destructive read endurance of 10^5 ^cycles of the PF devices. (**b**) Retention characteristics of the PF devices at RT and 85°C.

Figure 9a shows stable resistance ratio with an excellent non-destructive read up to 10^5 ^times for the PF devices. No fluctuation can be detected in LRS (25 kΩ) and HRS (2.2 MΩ) values at a V_read _of +0.2 V. Figure 9b shows the retention characteristics of the PF devices at 500 μA with a program/erase voltage of ± 4 V. The data retention ability is quite remarkable for both at RT and 85°C. Extrapolated 10 years of data retention with a resistance ratio of > 10^2 ^at RT and > 10 at 85°C are obtained. It is expected that a single IrO_x_-ND in the Al_2_O_3_/IrO_x_-NDs/Al_2_O_3_/WO_x _stack can control the resistive switching characteristics in future.

## Conclusions

Formation polarity dependent ReRAM devices using IrO_x_/Al_2_O_3_/IrO_x_-NDs/Al_2_O_3_/WO_x_/W structure have been investigated. The devices are confirmed by HRTEM, EDX, and XPS analyses. The improved resistive switching parameters such as stable set and reset voltages, HRS/LRS with a acceptable ratio of > 10, long read endurance of 10^5 ^times, and extrapolated 10 years data retention at 85°C under PF are obtained owing to the filament controlled at the IrO_x_-NDs/oxygen-rich AlO_x _interface. Due to the high-charge trapping density, controllable O^2- ^ions migration and nano filament formation through the core-shell IrO_x_-ND under PF, the improvement of the resistive switching memory characteristics is evident and the switching mechanism is explained successfully. A large memory size of > 60 Tbit/sq in. can be obtained. It is expected that the scalability potential of this resistive switching memory device could be the diameter (approximately 1.3 nm) of a single IrO_x_-ND in future.

## Competing interests

The authors declare that they have no competing interests.

## Authors' contributions

WB carried out the device fabrication, measurement, and data analysis under the instruction of SM. CSL helped to WB for experiments. YYC performed the TEM measurements of MIS capacitors under the instruction of JRY and SM. TCT performed the XPS measurements. HYL, WSC, FTC, MJK, and MJT performed via structure design and fabrication. All the authors contributed to the preparation and revision of the manuscript, and approved its final version.
